# Read All of Them

**Published:** 1919-01

**Authors:** 


### Read All of Them.

It is hardly necessary to call the attention of men as progressive as modern physicians are to the importance of reading the advertising pages of a medical journal if they would keep up with the progress of the profession. The advertisers furnish a large part of the equipment with which physicians must work. The manufacturing druggists, for instance, keep a corps of able physicians and chemists at work compounding and testing new remedies. Their advertisements, carried only in medical journals, are the announcements of the results of their investigations.

The physician who ignores the announcements of manufacturing chemists proclaims himself a back number. The surgeon who does not keep informed as to the improvements in surgical appliances had better retire from the practice.

In addition to these classes of advertising, manufacturers generally are learning that the best place to advertise other things used by physicians is in their medical journal, which has a closer place in their thoughts than other publications.

Naturally a man is most interested in a publication devoted to his profession, in which he employs his time and from which he makes his living, and especially if he thinks enough of that publication to subscribe and pay for it voluntarily.

Advertisers may feel pretty well assured that their advertisements in The Texas Medical Journal are read by progressive physicians. If there are subscribers who do not read them regularly they should pinch themselves to ascertain if they are really **live** physicians.

---

Let us invest a few dollars in our humanitarianism February 3-10, the week of the Armenian Relief Campaign.

